# Exploring the Thermal Degradation of Bakelite: Non-Isothermal Kinetic Modeling, Thermodynamic Insights, and Evolved Gas Analysis via Integrated In Situ TGA/MS and TGA/FT-IR Techniques

**DOI:** 10.3390/polym17162197

**Published:** 2025-08-12

**Authors:** Gamzenur Özsin

**Affiliations:** Department of Chemical Engineering, Faculty of Engineering, Bilecik Şeyh Edebali University, 11230 Bilecik, Turkey; gozsin@anadolu.edu.tr or gamzenur.ozsin@bilecik.edu.tr

**Keywords:** thermal decomposition, bakelite, in-situ evolved gas analysis, kinetics, thermodynamics, pyrolysis

## Abstract

Thermogravimetric analysis (TGA) is a key technique for evaluating the kinetics and thermodynamics of thermal degradation, providing essential data for material assessment and system design. When coupled with Fourier-transform infrared (FT-IR) spectroscopy or mass spectroscopy (MS), it enables the identification of evolved gases and correlates mass loss with specific chemical species, offering detailed insight into decomposition mechanisms. In this study, TGA was coupled with FT-IR and MS to investigate the thermal degradation behavior of Bakelite, with the aim of evaluating its kinetic and thermodynamic parameters under non-isothermal conditions, identifying evolved volatile compounds, and elucidating the degradation process. The results showed that higher heating rates led to increased decomposition temperatures and broader dTG peaks due to thermal lag effects. The degradation proceeded in multiple stages between 220 °C and 860 °C, ultimately yielding a carbonaceous residue. The activation energy increased with conversion, particularly beyond 0.5, indicating a greater energy requirement as degradation progressed. Peak values at conversion degrees of 0.8–0.9 suggested enhanced thermal stability or changes in the dominant reaction mechanism. Detailed kinetic analysis revealed complex decomposition pathways with variable activation energies and a pronounced kinetic compensation effect. Thermodynamic analysis confirmed the endothermic nature of the process, with increasing energy demand and non-spontaneous degradation of the resulting char. TGA/FT-IR and TGA/MS analyses identified the release of several compounds, including CO_2_, water, formaldehyde, and phenolic derivatives, at distinct stages. This comprehensive understanding of Bakelite’s thermal behavior supports its optimization for high-temperature applications, enhances material reliability and safety, and contributes to sustainable processing and recycling strategies.

## 1. Introduction

Advancements in technology necessitate the use of specialized and functionalized materials, such as thermosetting polymers, which hold significant industrial importance. Among these polymers, phenol-formaldehyde resins are particularly notable for their extensive applications in polymer science, as they enhance the mechanical and chemical properties of materials due to their exceptional performance in adhesion, heat and flame resistance, and electrical insulation [1]. However, the thermal degradation behavior of phenolic resins poses limitations on their applications, as drastic pyrolysis or thermal degradation can lead to the disintegration of polymeric structures, resulting in the failure of the resin matrix and its composites [2]. Heat treatment via pyrolysis, which involves thermal degradation under an inert atmosphere, decomposes thermoplastics, thermosets, and their composites into lower-weight molecules (liquid, gas, and solid fractions). The analysis of thermal degradation parameters offers critical insights into the internal structural dynamics of these polymeric materials [3,4,5]. Moreover, the thermal decomposition of polymeric matrices, such as formaldehyde resins, in the absence of air is a critical process in the production of carbon or related composites. This process involves the conversion of the resin matrix into amorphous carbon, accompanied by the release of numerous gaseous products. Nevertheless, potential damage during this degradation is significant due to thermal stresses and high pressures from the release of gaseous species from the structure [6,7,8]. Therefore, it is beneficial to investigate the pyrolytic thermal degradation behaviors of polymeric and thermosetting resins to better understand their thermal degradation processes and optimize their applications.

Thermogravimetric analysis (TGA) is a widely adopted and frequently used method for determining material degradation characteristics and quantifying reaction kinetics parameters due to its high precision [9,10,11,12,13,14]. Using non-isothermal mass loss data derived from TGA, iso-conversional methods have been extensively employed to generate kinetic parameters, which enable further thermodynamic analysis of degradation processes [15,16]. Although numerous researchers have investigated the thermal decomposition of various thermoplastics and thermosets [17,18,19], relatively little research has focused on Bakelite. Bakelite is known to char upon heating, prompting several studies on its conversion into carbonaceous materials, fuels, and chemical feedstocks [20,21,22,23,24,25]. Furthermore, the growing body of published work on electrical waste pyrolysis has heightened the need to study Bakelite, a widely used thermosetting phenol-formaldehyde resin in electrical applications, particularly its degradation kinetics and transport phenomena under varying heating rates and formulations [26,27,28,29].

The integration of hyphenated analytical techniques with TGA enables real-time, in situ monitoring of evolved gases during thermal degradation. This synergistic approach provides insights into both the chemical composition and concurrent physical transformations in materials undergoing thermal decomposition, offering a mechanistic understanding of reaction pathways. By capturing time-resolved data, these methods facilitate the precise characterization of kinetic pathways and degradation mechanisms, which are crucial for optimizing thermal processing conditions to achieve tailored material properties [30,31,32,33,34]. Consequently, this study aims to investigate the thermal degradation behavior of Bakelite using a combined TGA/FT-IR/MS system. Despite extensive research on the thermal degradation behavior of diverse polymeric materials and resins, to the best of the authors’ knowledge, no published study has specifically examined the thermal degradation of Bakelite in conjunction with evolved gas analysis. Therefore, this study estimates the kinetic parameters of thermal degradation and analyses thermodynamic data as well as evolved gas compositions to provide a comprehensive understanding of Bakelite pyrolysis. By employing simultaneous Fourier transform infrared spectroscopy (FT-IR) and mass spectrometry (MS) alongside TGA, valuable insights were gained into the chemical structural changes occurring during the degradation process.

## 2. Materials and Methods

### 2.1. Material Preparation and Characteristics

The Bakelite (polyoxybenzylmethylene glycol anhydride) samples used in this study were sourced from a phenolic resin production facility in Istanbul, Turkey. The thermosetting phenol-formaldehyde resin, synthesized through polycondensation of phenol and formaldehyde, was obtained as a commercial powder. As-received Bakelite specimens were subjected to minimal preparation, comminuted using a cutting mill (Retsch, Haan, Germany) and fractionated through sieving to isolate particles within the 112–224 μm range. Particle size distribution was carefully controlled to ensure experimental reproducibility in the subsequent thermoanalytical measurements. Additionally, the particle size was maintained sufficiently small to keep the Biot number (Bi) below unity, thereby minimizing internal temperature gradients and ensuring the accuracy of the derived kinetic parameters. Elemental composition of the sample was determined using an elemental analyzer (LECO CHNS-O 628 Series, St. Joseph, MI, USA).

### 2.2. Thermoanalytical Measurements

The thermoanalytical investigation encompassed three interrelated parts: kinetic analysis, thermodynamic evaluation, and evolved gas characterization during the thermal degradation of Bakelite. To achieve this, the thermal decomposition of Bakelite was performed using a thermogravimetric analyzer (TGA, Setaram Labsys Evo, Ankara, Turkey) coupled with a Fourier transform infrared (FT-IR) spectrometer (Nicolet iZ10, Madison, WI, USA) and a mass spectrometer (MS, Pfeiffer, Pfeiffer Vacuum GmbH, Aßlar, Germany). Approximately 10 mg of the Bakelite sample were heated from room temperature to 1000 °C at a constant nitrogen flow rate of 20 mL/min in an alumina (Al_2_O_3_) crucible. The thermogravimetric program included a stabilization period of 30 min at 25 °C, followed by heating to 1000 °C. To ensure experimental consistency, the sample mass was kept nearly identical for each run, minimizing variations in heat and mass transfer. This approach reduced discrepancies in endothermic and exothermic effects, as well as the diffusion rates of evolved gases and thermal gradients.

The TGA measured the sample weight and its rate of weight loss as functions of temperature and time, while kinetic calculations were performed using linear heating rates ranging from 5 to 40 °C/min. This experimental setup provided detailed insights into the thermal behavior of Bakelite, enabling the accurate characterization of its degradation kinetics and dynamic gas evolution under controlled conditions. During thermal degradation, the evolved gases were rapidly transported to the FT-IR gas cell via a heated transfer line, effectively preventing condensation of volatile compounds and dissociation of thermally unstable species. Pure nitrogen was used as the carrier gas throughout the system. Both the transfer line and the gas cell were maintained at 250 °C to ensure the integrity of the gas stream. Within the gas cell, time-resolved FT-IR spectra were continuously recorded. The instrument automatically performed wavenumber calibration using an internal He-Ne laser and applied background correction based on a pre-run reference spectrum. The resulting interferograms were Fourier-transformed into spectral vectors and compiled into a three-dimensional dataset representing absorbance as a function of wavenumber and temperature. All subsequent spectra were ratioed against the background to yield accurate absorbance values. The interface software synchronized the TGA temperature and time data with the FT-IR spectral acquisition, enabling real-time correlation between the thermal decomposition behavior and the chemical nature of the evolved gases. FT-IR spectra were recorded over the wavenumber range of 4000–400 cm^−1^ at a resolution of 4 cm^−1^. Simultaneous gas analysis was conducted by connecting the TGA furnace chamber outlet to the MS via a heated capillary column, which was maintained at 200 °C. A secondary electron multiplier (SEM)-type detector was used during simultaneous measurements. Initially, gas product intensities were analyzed using evolution curves obtained from a preliminary scan. Subsequently, evolved gases were scanned simultaneously for selected compounds using multiple ion detection (MID) during replicate experiments.

### 2.3. Kinetic Analysis

Understanding the thermal degradation of polymeric materials and resins is crucial for planning and optimizing industrial processes, as a thorough kinetic description can be effectively applied to inform process design and optimization. There are numerous studies on pyrolysis kinetics that illustrate various mechanisms based on different assumptions [35,36]. However, the fundamental rate equation for solid-state thermal conversion processes assumes that the conversion rate is proportional to the reactant concentration and dependent on temperature. Under a linear temperature heating rate (*β*), the kinetic expression is defined by two independent functions: the temperature function (*k*(T)) and the fractional conversion function (f(*α*)), as shown in Equation (1).

(1) $$\frac{d\alpha }{dt}=\beta \frac{d\alpha }{dT}=k(T)f(\alpha ).$$

The dependency of the rate constant, k, on temperature is described by the Arrhenius equation:

(2) $$k(T)=Aexp\left(-\frac{E_{a}}{RT}\right),$$

where *E* (or *E_a_*) is the activation energy, A the pre-exponential factor, and *R* the gas constant. By combining Equations (1) and (2), the reaction rate can be provided in the following form:

(3) $$\beta \frac{d\alpha }{dT}=A\exp\left(-\frac{E_{a}}{RT}\right)f(\alpha ).$$

On the other hand, fractional conversion is written as follows:

(4) $$\alpha =\frac{w_{o}-w_{t}}{w_{o}-w_{f}},$$

where *w_o_*, *w_t_*, and *w_f_* are sample masses presented at initial, arbitrary, and final times, respectively.

Equation (3) can also be integrated into the following:

(5) $$\int_{0}^{\alpha }\frac{d\alpha }{f(\alpha )}=g(\alpha )=\frac{A}{\beta }\int_{To}^{T}\exp\left(-\frac{E_{a}}{RT}\right)dT\equiv \frac{AE_{a}}{\beta R}p(u),$$

where *g*(*α*) and *p*(*u*) are known as the integrated form of the fractional conversion function *f*(*α*) and the temperature integral, respectively. *p*(*u*) is provided by the following:

(6) $$p(u)=\int_{\infty }^{u}-\frac{\exp(-u)}{u^{2}}du,$$

where *u* = *E_a_*/*RT*. Since *p*(*u*) has no analytical solution, it can be approximately represented via different empirical interpolation formulas such as Doyle [37], Agrawal–Sivasubramanian [38], and Senum–Yang [39] approximations. According to the assumed reaction mechanisms, there exist two possible approaches in kinetic analysis: iso-conversional (model-free) and model-fitting (or model-based).

Due to the numerous heterogeneous processes occurring during the conversion process, determining the pyrolysis kinetics remains a challenging task, and the selection of an appropriate kinetic model continues to be a topic of debate within the scientific community [40]. It is widely acknowledged that the improper application of model-based methods can lead to misleading results, particularly for systems involving multiple reaction mechanisms. However, iso-conversional methods have demonstrated their ability to provide model-independent estimates of activation energy, yielding meaningful values across a wide range of conditions. Iso-conversional methods are widely employed to determine activation energy as a function of the degree of conversion without requiring prior assumptions about the kinetic model governing the reaction. These methods are grounded in the iso-conversional principle, which states that the reaction rate at a constant degree of conversion depends solely on temperature. This principle can be readily demonstrated by taking the logarithmic derivative of the reaction rate equation (Equation (1)) at a constant degree of conversion.

(7) $${\left[\frac{\partial \ln(d\alpha /dt)}{\partial T^{-1}}\right]}_{\alpha }={\left[\frac{\partial \mathrm{lnk}(T)}{\partial T^{-1}}\right]}_{\alpha }+{\left[\frac{\partial \mathrm{lnf}(\alpha )}{\partial T^{-1}}\right]}_{\alpha },$$

where the subscript *α* indicates iso-conversional values, i.e., the values related to a given extent of conversion. Because at a constant conversion degree, *f*(*α*) is also constant, and the second term on the right-hand side of Equation (7) is zero. Thus,

(8) $${\left[\frac{\partial \ln(d\alpha /dt)}{\partial T^{-1}}\right]}_{\alpha }=-\frac{E_{a}}{R}.$$

In this study, triplicates of TGA experiments at four heating rates of 5, 10, 20, and 40 °C/min were carried out for better predictions of the activation energy, and five different model-free methods were used, namely Friedman [41], Flynn–Wall–Ozawa (FWO) [42,43], Kissinger–Akahira–Sunose (KAS) [44,45], Starink [46], and Tang [47], in order to determine the activation energy as a function of the conversion degree. The equations of all iso-conversional methods are summarized in Table 1.

### 2.4. Thermodynamic Analysis

The thermodynamic parameters of the process, including the enthalpy change (Δ*H*), Gibbs free energy change (Δ*G*), and entropy change (Δ*S*), were determined using the Eyring method after the determination of the activation energy [48,49] and pre-exponential factor (*A*) with the help of the equations provided below:

(9) $$A=\left[\beta E_{a}\exp\left(\frac{E_{a}}{RT_{m}}\right)\right]/(RT_{m}^{2}),$$

(10) $$\Delta H=E_{a}-\mathrm{RT},$$

(11) $$\Delta G=E_{a}+RT_{m}\ln\left(\frac{K_{B}T_{m}}{hA}\right),$$

(12) $$\Delta S=\frac{\Delta H-\Delta G}{T_{m}},$$

where *T_m_* is the maximum temperature peak observed from the dTG curve, *K_B_* is the Boltzmann constant (1.381 × 10^−23^ J/K), and *h* is the Planck constant (6.626 × 10^−34^ Js).

## 3. Results and Discussion

### 3.1. Elemental Composition of the Material and Analysis of TG/dTG Thermograms

Prior to the thermogravimetric analysis, the mass percentages of carbon (C), hydrogen (H), nitrogen (N), and oxygen (O) in Bakelite were determined using an elemental analyzer, as summarized in Table 2. This analysis provides precise quantification of the elemental composition, enabling verification of the material’s identity and detection of possible impurities. The elemental analysis of Bakelite reveals a composition consistent with its phenol-formaldehyde resin structure, featuring a high carbon content of 75.4%, which is typical of aromatic polymers. The moderate hydrogen content of 5.5% reflects the presence of methylene bridges and hydroxyl groups. Notably, the presence of 2.2% nitrogen suggests potential impurities or additives, as phenol-formaldehyde resins typically do not contain nitrogen. The oxygen content of 16.9% aligns with the hydroxyl groups and methylene bridges in the polymer. The H/C ratio of approximately 0.87 indicates a relatively low hydrogen content, while the O/C ratio of about 0.17 suggests a low oxygen content, consistent with Bakelite’s hydrophobic nature and resistance to moisture.

**Figure 1:**
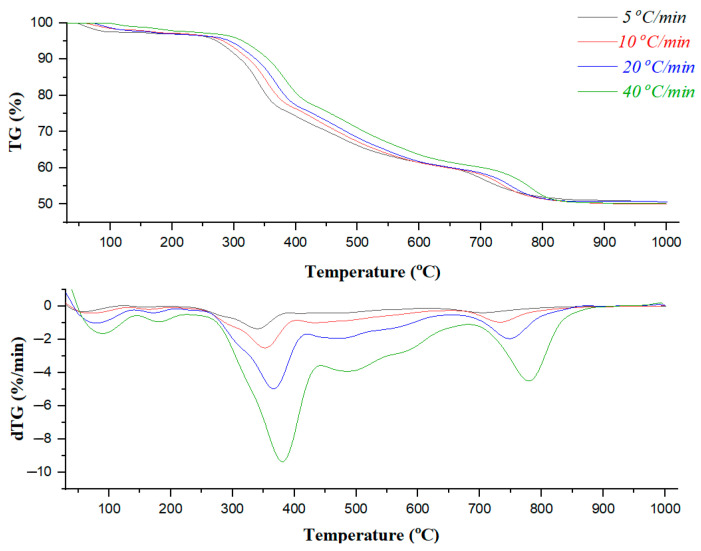
TG and dTG thermograms of Bakelite at different heating rates.

To gain a comprehensive understanding of the thermal degradation process, TGA was performed under a nitrogen atmosphere to examine the influence of heating rate variations, as reflected in the resulting thermograms. As illustrated in Figure 1, the TG curves maintain their overall shape regardless of heating rate variations; however, the mass loss temperatures shift to higher values at higher heating rates. This suggests that similar mechanisms are involved across different heating rates [50,51].

The thermogravimetric (TG) and derivative thermogravimetric (dTG) curves showed a noticeable shift of decomposition events toward higher temperatures as the heating rate increased. This shift manifested as a lateral displacement of peaks and shoulders, primarily attributed to thermal lag stemming from combined limitations in heat and mass transfer. It is well known that heating rate plays a critical role in governing the internal thermal distribution of the sample: at lower heating rates, heat penetrates the sample more uniformly, allowing efficient thermal conduction from the surface to the core. In contrast, at elevated heating rates, thermal gradients intensify, leading to inefficient heat transfer and delayed decomposition in interior regions. This phenomenon is characterized by thermal hysteresis, where the degradation temperature for a given degree of conversion increases with heating rate. Thermal hysteresis leads to broader dTG peaks, indicating a more gradual and less synchronized decomposition process due to microstructural resistance to heat propagation. At low heating rates, the system permits more time for the sample and purge gas to thermally equilibrate, resulting in an earlier onset of decomposition. Conversely, at high heating rates, the residence time within a given temperature range is reduced, requiring higher temperatures to initiate comparable levels of degradation. Furthermore, decomposition products may accumulate on the outer surfaces of Bakelite particles, reducing diffusion efficiency and further influencing degradation behavior by hindering mass transport from the particle interior [52,53]. These heating rate-dependent effects reflected the dynamic thermal response of Bakelite’s crosslinked, aromatic microstructure under varying thermal stresses. Consequently, the characteristic curve shifts toward higher temperatures.

The pyrolysis of polymers typically progresses through several distinct stages, including melting (solid to liquid), pyrolysis characterized by the evaporation of volatiles (liquid to gas), and coke formation at the final stage. According to various studies, the pyrolysis of phenol-formaldehyde resins follows three primary degradation stages. The first stage involves crosslink formation due to condensation reactions between unreacted functional groups in the cured resin, producing water and heavier aromatic species. This stage occurs within the temperature range of 550–800 K (~277–527 °C). The second stage, occurring between 700–1100 K (~427–827 °C), involves the breaking of crosslinks, leading to the formation of degradation byproducts, such as methane, hydrogen, carbon monoxide, small oligomers, and water, due to structural breakdown. The final stage, above 850 K (~577 °C), involves the charring of the remaining resin through hydrogen gas release as hydrogen atoms are removed from the aromatic ring structure. During this stage, the resin network collapses, followed by carbonization and graphitization processes [54,55,56,57,58,59,60].

The pyrolysis characteristics of Bakelite were determined from TG and dTG profiles, including initial peak temperature (Ti), maximum peak temperature (T_p_) and final peak temperature (T_f_) in the main pyrolysis zone, as summarized in Table 3. Peak decomposition temperatures corresponding to heating rates of 5, 10, 20, and 40 °C/min were recorded at 343.7 °C, 351.7 °C, 366.4 °C, and 379.2 °C, respectively. Additionally, reactivity increased from 0.14 to 0.87%/min·mg as the heating rate was elevated from 5 to 40 °C/min. This enhancement in reactivity with increasing heating rate can be attributed to the acceleration of thermal degradation, more rapid mass loss, and a shift in decomposition kinetics toward higher temperatures. Elevated heating rates improve thermal reactivity in TGA by promoting faster reaction rates, reducing thermal lag and internal heat transfer limitations, and potentially altering the dominant reaction pathways within the constrained experimental time window. At higher heating rates, Bakelite absorbed thermal energy more rapidly, facilitating bond cleavage and increasing the rate of degradation. This resulted in greater mass loss per unit time and a sharper thermal response. In contrast, at lower heating rates, the sample remains at intermediate temperatures for extended durations, enabling a more gradual and sequential decomposition process. The more rapid attainment of elevated temperatures at higher heating rates also leads to increased volatile release, contributing to the observed rise in reactivity. On the other hand, the thermal degradation of Bakelite actively commenced at temperatures above 220 °C. Below this threshold, minor weight loss occurred due to evaporation of residual moisture and low-boiling-point volatiles trapped within the Bakelite matrix without significant structural degradation. Major weight loss occurred up to approximately 450 °C, marked by rapid mass loss corresponding to the breakdown of the crosslinked phenol-formaldehyde polymer network. This phase was characterized by prominent peaks in the dTG curves reflecting maximum decomposition rates that dependend on the heating rate. Beyond 450–470 °C (depending on heating rate), mass loss slowed significantly as a thermally stable carbonaceous residue remained. After approximately 850 °C, thermal degradation was nearly complete with char yields exceeding 50.1 wt.% across all heating rates. A combination of Gaussian and Lorentzian peak functions was employed as a Voigt function to deconvolute the dTG thermogram within the initial and final temperatures of thermal degradation, aiming to analyze the complex characteristics of the process at a heating rate of 40 °C. The highest heating rate was chosen for the deconvolution process to enhance peak resolution and minimize peak overlap, making it more suitable for complex thermal decomposition studies. This approach effectively reduced peak broadening, improved the differentiation of overlapping reactions, and increased the signal-to-noise ratio, while also minimizing secondary reactions, such as char formation and volatile recombination, that can distort the dTG signal at lower heating rates.

**Figure 2:**
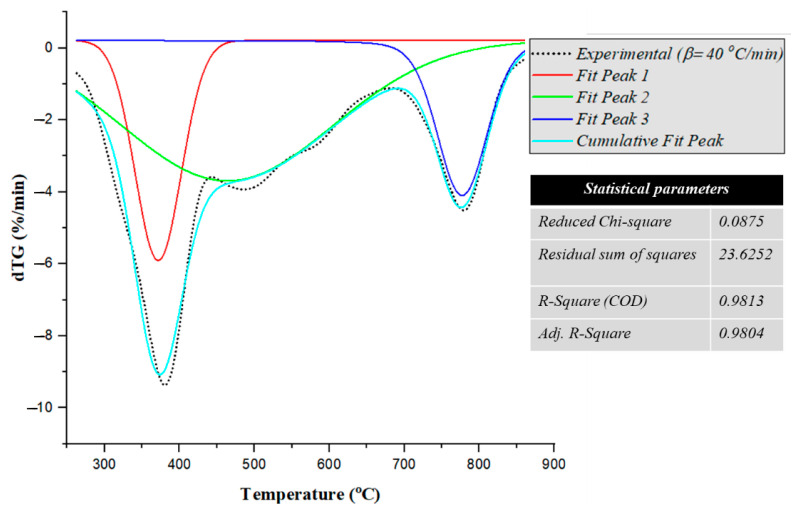
Deconvolution of the experimental dTG curve.

The deconvoluted curves are presented in Figure 2. Utilizing a non-linear curve-fitting approach, three distinct peaks were identified. The high R^2^ value of 0.9813 and the adjusted R^2^ of 0.9804 indicate that the model explains approximately 98% of the variance in the data, suggesting a highly accurate fit. Furthermore, the reduced chi-square value of 0.0875 implied relatively small residuals, further confirming the robustness of the model. The residual sum of squares (RSS), calculated as 23.6252, supports the reliability of the fitted peaks by demonstrating minimal discrepancies between the model and the experimental data. The presence of three distinct peaks suggested that the overall curve represents multiple overlapping thermal events, likely corresponding to different degradation steps. Among these, Peak 2, which exhibited the highest amplitude and broadest width, appears to dominate the thermal decomposition process, indicating a major degradation phase.

### 3.2. Kinetic Analysis Results

**Figure 3:**
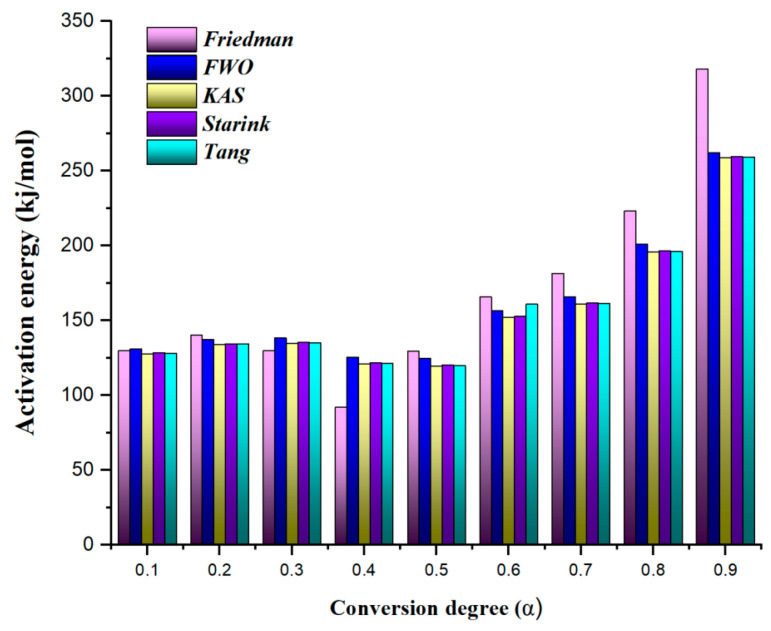
Activation energy distribution during thermal degradation.

As detailed in the kinetic analysis section, five distinct iso-conversional kinetic methods (Friedman, FWO, KAS, Starink, and Tang) were employed to analyze experimental TGA data. This analysis aimed to elucidate the variation in activation energy as a function of the degree of conversion. The activation energy values corresponding to conversion levels spanning from 0.1 to 0.9 are illustrated in Figure 3. A systematic comparison of the results derived from the different iso-conversional models was conducted.

The results indicate that the activation energy varied significantly with the degree of conversion, showing a clear increasing trend, particularly beyond a conversion degree of 0.5. This indicates that as degradation progresses, greater energy is required to decompose the remaining material. In the early stages, degradation predominantly involves the cleavage of weaker and more accessible bonds, such as side chains or less crosslinked segments, which require relatively low activation energy. As degradation progresses, the remaining molecular structure becomes increasingly enriched in highly crosslinked, aromatic segments. These regions of the polymer network are known to involve strong covalent bonds, which are more resistant to thermal cleavage compared to the initially decomposed, less stable structures. Consequently, the breakdown of these robust chemical linkages demands higher activation energy, leading to an increase in the activation energy. The highest activation energy values were observed at higher conversion degrees (0.8–0.9), implying that the degradation process becomes more energy-intensive in its later stages. This increased energy requirement is likely due to the formation of thermally stable residues or changes in the dominant reaction mechanism. Fluctuations in the activation energy values suggest a multi-step degradation mechanism for Bakelite, with a possible transition between reaction mechanisms occurring around 0.4–0.5. The average activation energy values differed across the various iso-conversional models, reflecting inherent differences in their assumptions and computational approaches. Among these methods, the Friedman model yielded the highest average activation energy at 167.7 kJ/mol, consistent with its model-free nature, which does not assume a constant reaction model, thereby making it more sensitive to kinetic variations. The FWO method followed with an average of 160.2 kJ/mol, while the KAS, Starink, and Tang methods produced slightly lower yet comparable values of 155.9 kJ/mol, 156.6 kJ/mol, and 157.3 kJ/mol, respectively. Although all methods exhibited similar trends, the Friedman method consistently reported higher activation energy values, particularly at higher conversion degrees. In contrast, the FWO, KAS, Starink, and Tang methods followed similar activation energy trends but generally yielded lower values. Notably, differences between these models remained relatively small at lower conversion degrees (0.1–0.3) but became more pronounced at higher conversion levels. This indicates that the choice of kinetic model plays a more significant role in activation energy estimation as the degradation process advances, particularly in the later stages.

In chemical kinetics, the pre-exponential factor (A) represents the frequency of molecular collisions among reactants. Its variation with respect to the conversion degree provides critical insights into the reaction mechanism, kinetic complexity, and energy barrier fluctuations during the decomposition process. In general, a low pre-exponential factor (<10^9^ s^−1^) is recognized as characteristic of surface reactions in reaction kinetics. When the reaction does not depend on surface area, such a reduced pre-exponential factor is typically interpreted as indicating a tight junctional complex, commonly referred to as a closed complex. In contrast, a high value of A (≥10^9^ s^−1^) suggests a loose simple complex, also termed a junctional complex. Moreover, larger pre-exponential factors signify heightened sensitivity within a given reaction temperature range [61]. The numerical values of the pre-exponential factors are presented in Table 4. Accordingly, the pre-exponential factor values exhibited significant variation across different conversion degrees, highlighting a complex kinetic behavior in the thermal degradation process. At low to moderate conversion levels (0.1–0.5), the pre-exponential factor remained relatively low, fluctuating between 1.37 × 10^4^ s^−1^ and 2.72 × 10^7^ s^−1^, with a notable drop at 0.4 (1.37 × 10^4^ s^−1^), which may have indicated a shift in the reaction mechanism or a phase transition in thermal degradation. As the conversion degree increased (0.6–0.8), a rapid rise in A was observed, reaching 3.70 × 10^10^ s^−1^ at 0.6 and further escalating to 1.19 × 10^15^ s^−1^ at 0.8, suggesting a transition in the reaction mechanism that required a significantly higher frequency of molecular collisions. At the extreme conversion level (0.9), the highest pre-exponential factor value, 3.74 × 10^23^ s^−1^, was recorded, indicating a diffusion-controlled or residue-driven process in the final degradation stages. The overall analysis of the data, with an average pre-exponential factor of 4.15 × 10^22^ s^−1^, and considerable fluctuations in the level of molecular collisions indicated a multi-stage degradation mechanism, as can be concluded from the activation energy distribution. The sharp increase at higher conversion levels implied a transition from a kinetically controlled process to a diffusion-limited or char-formation phase, a common phenomenon in polymer degradation. The extreme value observed at 0.9 suggested the presence of highly resistant char residue, requiring significantly more energy and molecular interactions to decompose. The linear correlation between the natural logarithm of the pre-exponential factor (lnA) and the activation energy (E_a_) in facilitating the thermal decomposition of Bakelite is also depicted in Table 4. The regression analysis of the compensation plot yielded a high regression coefficient (R^2^ > 0.99), indicating a strong linear relationship. In other words, the activation energy and pre-exponential factor exhibit a well-defined linear dependence, which can be described by a mathematical expression of the form *lnA* = *aEa* + *b*, representing an energy compensation effect [62]. Based on the results, this relationship was determined to be *lnA* = 0.0197*Ea* − 8.5125 for the thermal decomposition process.

### 3.3. Thermodynamic Analysis

Following the kinetic analysis, which primarily focused on the activation energy and the pre-exponential factor as indicators of the reaction rate, thermodynamic evaluation provides a complementary perspective on the thermal degradation behavior. In TGA-based studies, understanding the interrelationship between enthalpy change (ΔH), Gibbs free energy change (ΔG), and entropy change (ΔS) is essential for elucidating not only the reaction mechanism but also the thermodynamic feasibility and the nature of the transition state. While activation energy reflects the kinetic barrier, ΔH, ΔG, and ΔS offer deeper insights into the energetic favorability and molecular disorder accompanying the decomposition process. Assessing these parameters allows for a more comprehensive interpretation of the system’s thermal stability and supports the identification of suitable operating conditions for thermally driven reactions. The interplay of thermodynamic parameters (ΔH, ΔG, and ΔS) captures the balance between the energy input needed to cleave bonds (ΔH), the increase in disorder resulting from bond cleavage (ΔS), and the overall feasibility of the reaction (ΔG). Changes in these thermodynamic values are thus directly tied to the nature and extent of chemical bond breaking and formation throughout the thermal degradation process.

**Figure 4:**
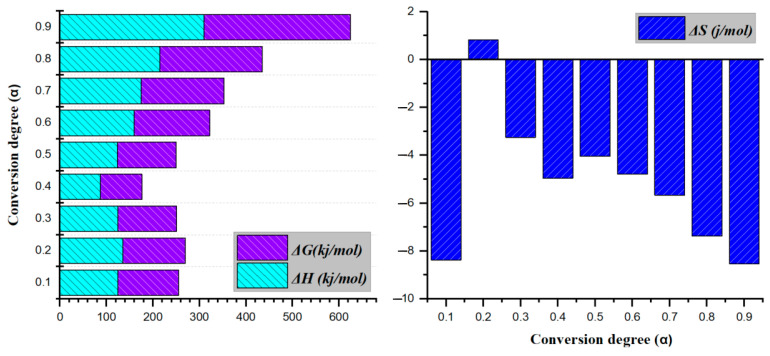
Thermodynamic parameters across different conversion levels during the thermal degradation of bakalite.

Accordingly, thermodynamic parameters were calculated to further characterize the decomposition process and its driving forces. For clarity, Figure 4 illustrates plots of thermodynamic functions, highlighting the relationships among ΔH, ΔS, and ΔG as a function of the conversion degree, which ranges from 0.1 to 0.9.

Enthalpy measures the energy required for a chemical reaction to transform reactants into products, and it is well established that a small difference between the activation energy and ΔH favors the formation of an activated complex [63,64,65]. In other words, ΔH corresponds to the net energy absorbed or released during bond breaking and formation. Since breaking covalent bonds is endothermic, ΔH is generally positive during degradation, reflecting the energy input required to cleave chemical bonds in the polymer matrix. The enthalpy changes for the thermal degradation of Bakelite remained within a moderate range (approximately 86.5–174.6 kJ/mol) for most of the conversion process, with a distinct dip at 0.4 (86.5 kJ/mol) and a marked increase at higher conversions (0.8–0.9), ultimately reaching 309.6 kJ/mol at 0.9. The average enthalpy change was determined to be 161.5 kJ/mol. This trend indicates that the thermal degradation of Bakelite becomes more energy-intensive at elevated conversion levels, possibly reflecting a shift in decomposition pathways or reaction mechanisms. Positive ΔH values during the conversion indicate the endothermic nature of the process, thereby requiring an external energy input for product formation.

ΔG dictates the spontaneity of the degradation reaction and quantifies the net energy exchange associated with the formation of a transient intermediate complex during bond dissociation in reactant molecules. This process reflects the thermodynamic driving force of chemical reactions, where bond reorganization in reactants leads to energy release or absorption [66]. Across the entire range of conversion (0.1–0.9), ΔG values remained positive, indicating that the process is non-spontaneous under the given conditions and requires external energy input. From 0.1 to 0.3, ΔG stayed around 130 kJ/mol, briefly declined to 89.6 kJ/mol at 0.4, then increased significantly from 0.6 onward, reaching a maximum of 314.9 kJ/mol at 0.9. This trend suggests that thermal degradation becomes progressively less thermodynamically favorable at higher conversions, likely reflecting changes in reaction pathways or constraints as degradation proceeds. In the final stage of thermal degradation, the remaining material often consists of more stable, carbon-rich fractions, which require greater energy input to decompose. Under an inert atmosphere, the absence of oxidizing agents precludes pathways that might otherwise facilitate further breakdown or energy release. Consequently, the process becomes increasingly unfavorable at advanced conversion levels, causing a rise in ΔG.

In thermal decomposition reactions, entropy change (ΔS) measures the extent of molecular disorder. A negative ΔS value signifies a transition from a more disordered state to a more ordered one. Additionally, ΔS represents the uncertainty associated with the process at each decomposition stage. Notably, small ΔS values indicate that the decomposition process involves minimal physical and chemical changes, suggesting that the system operates close to thermodynamic equilibrium [67,68,69]. Thermal degradation usually involves breaking down large, ordered macromolecules into many smaller molecules, gases, or volatiles, thereby significantly increasing entropy. This positive ΔS drives the reaction forward thermodynamically. Based on calculations of Bakelite degradation, ΔS values were found to be negative except for a small positive value near a conversion degree of 0.2. In general, a negative ΔS suggests a net decrease in the system’s entropy, i.e., a transition toward a more ordered state, which is consistent with the formation or reorganization of more stable structures as the material thermally degraded in an inert nitrogen environment, the absence of oxidizing reactions prevents further fragmentation or substantial gaseous byproduct formation, thereby limiting disorder. Consequently, as conversion advances, the decomposition pathway increasingly favors char and other condensed-phase structures, ultimately reducing the system’s overall entropy. The minor positive ΔS at a conversion of 0.2 reflects a transient phase in which certain functional groups or volatile components are released, temporarily increasing molecular disorder before the system settles into more stable, lower-entropy arrangements. Overall, the predominantly negative ΔS values indicated that the dominant decomposition mechanism of Bakelite involved processes that create more ordered residues, rather than generating large amounts of gaseous products or highly disordered intermediates. A negative average ΔS value (−5.1 J/mol.K), which indicates that the overall disorder of the system decreases during decomposition. Although fragmentation may occur during Bakelite’s thermal degradation, the overall decrease in entropy can be attributed to the formation of more structurally ordered char, along with the release of gas-phase species.

### 3.4. Evolved Gas Analysis Using Simultaneous In Situ TGA/FT-IR and TGA-MS Analyses

Evolved gas analysis is typically performed by integrating thermogravimetry with complementary analytical methods, allowing for a comprehensive understanding of the decomposition process and the identification of evolved gases [70,71]. By analyzing characteristic functional groups and covalent bonds in real time, TGA/FT-IR can detect key decomposition products, enabling the identification of functional group removal and permanent gas release during thermal treatment [72]. Meanwhile, TGA/MS provides a temperature-resolved profile of the evolving gases by ionizing them and detecting their mass-to-charge (*m*/*z*) ratios. Combining TGA/FT-IR and TGA/MS in a single instrument allows the comprehensive, real-time monitoring of thermal decomposition, simultaneously capturing both weight changes and gas-phase information (IR spectra and *m*/*z* profiles). This dual detection not only enhances accuracy in identifying evolved species and clarifies overlapping signals but also ensures optimal correlation by analyzing the same sample under identical conditions, making it particularly valuable for studying complex thermal reactions or multi-step decomposition processes.

**Figure 5:**
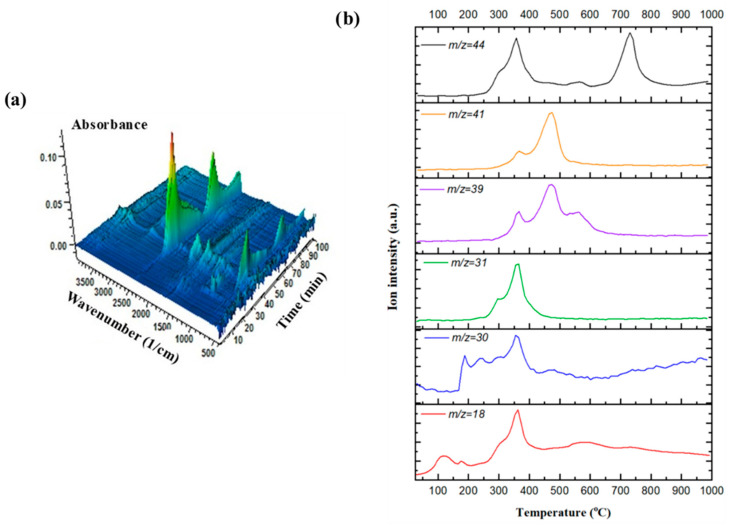
Three-dimensional TGA/FT-IR spectrum and single-ion current curves monitored (**a**) and obtained from simultaneous TGA/MS coupling (**b**).

Figure 5a displays a three-dimensional FT-IR spectrum acquired during the thermal decomposition of Bakelite. This 3D representation is particularly useful for monitoring the absorbance associated with the vibrational modes of various chemical bonds and functional groups in the gases evolved within the TGA furnace throughout the entire thermal degradation process. Due to the direct proportionality between spectral absorbance at a specific wavenumber and the concentration of gaseous species, as described by Beer–Lambert’s law, absorbance measurements can serve as a valuable tool for analyzing gas evolution profiles [73]. According to the diagram, the appearance of absorbance peaks seemed to align closely with the mass loss observed in the dTG curve. In the FT-IR spectrum, the strong asymmetric stretch between 2250 and 2500 cm^−1^ and the bending mode between 580 and 730 cm^−1^ indicated the release of CO_2_. The high intensity of these peaks suggested that CO_2_ is the dominant evolved gas, likely released in multiple stages due to the complex kinetic pathways of Bakelite degradation. The broad O–H stretching band in the range of 3500–3100 cm^−1^ and the H–O–H bending mode at around 1650 cm^−1^ signified water evolution during the thermal decomposition of Bakelite. A C=O stretch at about 1720 cm^−1^, along with additional fingerprint modes between 1500 and 1100 cm^−1^, pointed to the evolution of formaldehyde (HCHO). Another asymmetric stretch at around 2350 cm^−1^ and the bending mode at ~670 cm^−1^ are also reported to indicate minor CO release during thermal degradation. Aromatic C–C ring stretches in the 1500–1600 cm^−1^ region, a broad O–H stretch from 3500–3200 cm^−1^, and out-of-plane ring bends between 700–900 cm^−1^ are associated with the release of phenolics. Minor absorbance bands within the 2850–3030 cm^−1^ region correspond to C–H stretching vibrations, primarily arising from the presence of light hydrocarbon gases.

TGA/MS offers comprehensive insights into thermochemical processes by enabling precise, real-time monitoring of evolving gases. When combined with TGA/FT-IR, this approach provides enhanced molecular-level information, such as detailed molecular weight identification, effectively addressing the challenges posed by overlapping signals commonly encountered in FT-IR spectra due to similar vibrational modes. Moreover, MS exhibits a broader detection capability, successfully identifying compounds that may lack distinct IR-active functional groups, which is a limitation inherent to FT-IR techniques. While TGA/FT-IR remains highly valuable for functional group characterization and qualitative insights into molecular structures, TGA-MS excels in generating precise quantitative data on gas evolution, rendering it particularly advantageous for detailed thermochemical investigations [74]. Therefore, a comparative TGA/MS analysis was performed simultaneously with TGA/FT-IR to identify specific compounds released during the thermal degradation of Bakelite. Figure 5b illustrates the MS signals corresponding to selected chemical groups, which were monitored after an initial scan and recorded concurrently with FT-IR data. Single-ion monitoring curves were obtained for volatile compounds produced during Bakelite degradation, including H_2_O (water, *m*/*z* 18), CH_2_O^+^ (formyl cation from formaldehyde, *m*/*z* 30), CH_2_OH^+^ (hydroxymethyl cation from methanol, *m*/*z* 31), C_3_H_3_^+^ (propenyl cation from phenol, *m*/*z* 39), C_3_H_5_^+^ (allyl cation from phenol, *m*/*z* 41), and CO_2_ (carbon dioxide *m*/*z* = 44). The presence of peaks at different temperatures suggested distinct thermal decomposition stages, as also concluded from the kinetics.

Water evolved in multiple stages during thermal degradation due to dehydration reactions, likely originating from residual moisture trapped within the polymer and from the elimination of hydroxyl (-OH) groups in phenolic units. The intensity of the water signal diminished significantly beyond 400 °C, indicating that dehydration primarily occurred in the early stages of decomposition. The *m*/*z* 30 signal, corresponding to the formyl cation (CH_2_O^+^) derived from formaldehyde, displayed a broad peak between 300–500 °C, with residual intensity continuing at higher temperatures. Formaldehyde is a characteristic degradation product of Bakelite, primarily resulting from the cleavage of methylene (-CH_2_-) bridges linking phenolic units in the polymer structure. The gradual intensity reduction beyond 500 °C indicated that formaldehyde release predominantly occurred during the initial stages of thermal degradation. The *m*/*z* = 31 signal, representing the hydroxymethyl cation (CH_2_OH^+^) associated with methanol, exhibited a distinct peak between 350–450 °C. This ion likely arose from methanol (CH_3_OH) or protonated formaldehyde, formed through the cleavage of hydroxymethyl (-CH_2_OH) groups from phenolic units or through secondary formaldehyde reactions. The intensity diminished after 500 °C, suggesting most methanol evolved within the early to intermediate temperature range. The *m*/*z* = 39 signal, corresponding to the propenyl cation (C_3_H_3_^+^) derived from phenol, displayed multiple intensity fluctuations between 400–600 °C. This ion represented phenolic degradation fragments, likely generated by the cleavage of the phenolic backbone into smaller hydrocarbon fragments. The appearance of multiple peaks indicated overlapping degradation pathways, possibly involving crosslinking and rearrangement reactions. The *m*/*z* 41 signal, attributed to the allyl cation (C_3_H_5_^+^) from phenol, exhibited a pronounced peak near 500 °C. As a significant fragment from phenol decomposition, the higher intensity compared to *m*/*z* = 39 implied preferential formation of allyl-type structures during thermal degradation. The signal decreased sharply after 600 °C, signifying complete volatilization at higher temperatures. The *m*/*z* = 44 signal, commonly associated with carbon dioxide (CO_2_) or other oxygenated species, presented a broad peak between 500–800 °C with two distinct maxima. This ion likely arose from decarboxylation of residual oxygen-containing functional groups and secondary oxidation of degraded polymer fragments. The two-stage evolution suggested an initial release associated with phenolic degradation (500–600 °C), followed by oxidation of residual carbonaceous species at elevated temperatures (700–800 °C).

## 4. Conclusions

The thermal degradation of Bakelite under a nitrogen atmosphere was investigated using dynamic thermogravimetric analysis combined with online FT-IR and MS spectral analysis. At higher heating rates, the dTG curves shifted to higher decomposition temperatures due to thermal lag effects, resulting in broader peaks and delayed core decomposition. The peak decomposition temperature and reactivity increased linearly with the heating rate, driven by enhanced thermal energy absorption that accelerates bond cleavage and volatile release. Bakelite’s thermal degradation occurs in multiple stages, commencing at 220 °C and concluding around 860 °C, leaving a stable carbonaceous residue. Thermal degradation kinetics were analyzed using five iso-conversional methods, revealing complex decomposition pathways with varying activation energies and pre-exponential factors that reflect mechanistic shifts during the degradation process. A strong linear energy-compensation effect was also observed. Thermodynamically, the process is endothermic, as indicated by positive ΔH values, requiring external energy input and highlighting increasing energy demands as degradation progresses. ΔG values confirmed the non-spontaneous nature of the process, particularly for the degradation of carbon-rich char. Predominantly negative entropy ΔS values signaled increased molecular order, except for a transient rise at a conversion of 0.2, underscoring structural reorganization and char formation over gaseous byproducts. The three-dimensional TGA/FT-IR analysis revealed that thermal degradation involves the release of CO_2_, water, formaldehyde, CO, light hydrocarbons, and phenolic compounds. TGA/MS findings further emphasized the complex, multi-stage nature of Bakelite’s thermal decomposition by monitoring specific ion curves, which highlighted the release of these gases in distinct stages. Ultimately, elucidating Bakelite’s thermal decomposition kinetics, thermodynamics, and evolved gas analysis provided critical insights into its thermal stability and degradation behaviors, thereby optimizing its performance in high-temperature applications, enhancing reliability and safety, and informing optimal processing and recycling strategies that benefit both economic and environmental sustainability. Although this study provides a comprehensive analysis of Bakelite’s thermal degradation through TGA-based kinetic, thermodynamic, and evolved gas analyses, certain limitations, such as the use of a fixed atmosphere and the inherent assumptions in kinetic modeling, should be acknowledged. Future research should incorporate complementary techniques, while investigations on modified or recycled phenolic resins and Bakelite composites could further advance the development of thermally stable and sustainable materials for high-performance applications.

## Figures and Tables

**Table 1:** Equations of the kinetic models.

Friedman:	$\ln\left(\beta \frac{d\alpha }{dT}\right)=\ln A+\ln f(\alpha )-\frac{E_{a}}{RT}$.
FWO:	$\ln\beta =\ln\frac{AE_{a}}{Rg(\alpha )}-5.331-1.052\frac{E_{a}}{RT}$.
KAS:	$\ln\left(\frac{\beta }{T^{2}}\right)=\ln\left(\frac{AR}{E_{a}g(\alpha )}\right)-\frac{E_{a}}{RT}$.
Starink:	$\ln\left(\frac{\beta }{T^{1.8}}\right)=C_{s}-1.0037\frac{E_{a}}{RT}$.
Tang:	$\ln\left[\frac{\beta }{T^{1.894661}}\right]=\left[\ln\left(\frac{AE_{a}}{\beta R}\right)+3.63504095-1.894661\ln E_{a}\right]-1.00145033\left(\frac{E_{a}}{RT}\right)$.

**Table 2:** Ultimate analysis of Bakelite.

C (%)	75.4
H (%)	5.5
N (%)	2.2
* O (%)	16.9
H/C	0.87
O/C	0.17

* From difference.

**Table 3:** Characteristic pyrolysis temperatures.

β (°C/min)	T_i_ (°C)	T_p_ (°C)	T_f_ (°C)	R_p_ (%/min·mg)
5	219.4	343.7	849.2	0.14
10	222.0	351.8	855.6	0.16
20	260.5	366.4	858.0	0.47
40	262.6	379.2	862.2	0.87

T_i_ is the initial thermal decomposition temperature. T_p_ is the peak temperature in the main degradation zone. T_f_ is the terminal thermal decomposition temperature. R_p_ is the reactivity.

**Table 4:** Pre-exponential factor values and compensation plot.

α	A (s^−1^)	Kinetic Compensation Plot
0.1	2.73 × 10^7^	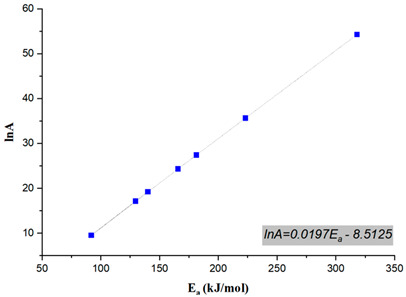
0.2	2.22 × 10^8^	
0.3	2.76 × 10^7^	
0.4	1.37 × 10^4^	
0.5	2.72 × 10^7^	
0.6	3.70 × 10^10^	
0.7	8.00 × 10^11^	
0.8	3.01 × 10^15^	
0.9	3.74 × 10^23^	
Average	4.15 × 10^22^	

## Data Availability

Part of the data is contained within the article. Further inquiries can be directed to the corresponding author.
